# First CytoJournal Peer-Reviewer's Retreat in 2006 – Open access, peer-review, and impact factor

**DOI:** 10.1186/1742-6413-3-5

**Published:** 2006-03-27

**Authors:** Vinod B Shidham, Lynn Sandweiss, Barbara F Atkinson

**Affiliations:** 1 Co-editor-in-Chief, CytoJournal, Medical College of Wisconsin, Milwaukee, WI, USA; 2 Chair- Open-Access advocacy for CytoJournal, University of California, Los Angeles, CA, USA; 3 Co-editor-in-Chief, CytoJournal, Kansas University Medical Center, Kansas City, KS, USA

## Abstract

CytoJournal organized its first Peer-Reviewer's Retreat of 2006 during the United States and Canadian Academy of Pathology Annual Meeting at Atlanta on Feb 12, 2006. The major topics discussed were open access, peer review, and impact factors. Representative participants volunteered to join the task force to prepare an instructional guide for peer-reviewing cytopathology manuscripts. Concern about the impact factor for CytoJournal was discussed. A feedback to its readers and authors was recommended. Impact factor calculation needs at least three years of journal statistics. It is only possible after two years from the time a journal is first accepted by Thomson-ISI for citation tracking. CytoJournal is still too new for an impact factor to be calculated. However, general progress of CytoJournal suggests an encouraging pattern for high impact factor.

## 

The authors presented and discussed with the participants about Open Access (Additional file 1) and the Peer-Review process (Additional file 2). At the end of the retreat, a task force for designing an instructional guide tailored specifically to cytopathology manuscripts was created. The model instructional guide from the American College of Emergency Physicians, discussed with the participants at the retreat, would be followed as a basic guideline. In addition, there was a discussion about the impact factor of CytoJournal.

## Open access

**Figure 1:**
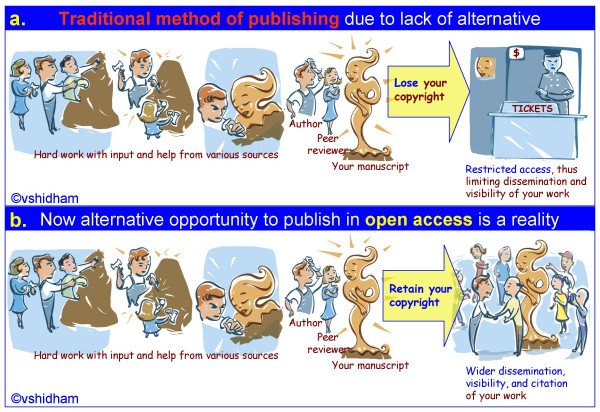
In the past an open access publication model was not available as option and the best possible avenue was the traditional model (a), which has served its purpose. With advances in web technology and digitization the open access with significant benefits to authors, readers, and ultimately the general public has become apparent. Open access (b) movement has been established successfully and we, in academia, should put all our energy behind it without prejudice from perceived or real pressures. ^©^vshidham. (Artist- Beatrice Favereau, bfavereau@sympatico.ca, )

We as academicians strive to create wonderful sculptures in the form of published research in the hope of sharing it with all our colleagues and the general public (Figure 1). With the traditional model for publishing scholarly work, we have to lose the copyright (and in reality the only right with reference to that work) and turn it to a close custody with restricted access (Figure 1a). Open access is now a reality and is widely appreciated and respected [1-5]. It does not need high tech deduction to understand the benefits and philosophical principles of open access (Figure 1b). However, we as authors and the general public have to be more proactive and imaginative to create a more robust sustainable model for generations to come. Traditional methods of publishing have done an excellent job with the resources and technology available at the time. Today, with all the advances in communications technology, digitization, internet, archiving, memory cost, and so on, it is high time to think and revolutionize our attitude towards the way we publish our work.

Whatever the form and mode of publication, we all work hard in any model. Our ultimate goal is disseminating and sharing the scholarly information, even at the cost of losing our copyright in traditional model of publishing (Figure 1a). In that model, only those of our colleagues who are fortunate enough to be able to pay for the journal access can read your publication. Is this restricted access what we want as a researcher? And if not, why should we follow such a flawed and unfavorable model? In the past the answer was simple – we did not have any alternative!

Now with advances in the web and digitization technology we have the great alternative of publishing in an open access journal (Figure 1b) [1-5]. But any new model, no matter how powerful and beneficial it may be, has to evolve on all fronts including financial. Success of any enterprise depends on its financial viability. Open access is showing tremendous success even on that front.

At this juncture, it is crucially important that all professional societies, associations, and funding agencies strongly consider supporting open access and extend opportunity to their constituents to publish their work in open access to further their ultimate mission of disseminating scientific information to fulfill their public responsibility. Most societies have their own journals. Many of these journals are trying to be open at both the readership and authorship ends, to benefit their membership and general public [6]. Other societies are just supporting private traditional non-open access journals owned by commercial publishers. We encourage all professional organizations to revisit this important issue and consider open access to steer the publishing scenario in the right direction, which would work for all of us.

What can we do as individual academicians? The first step would be to question the current publication model and insist on the open access model in our respective societies, including free publication in society journals for all their members. It could be argued that those members who are not interested in publishing may discontinue their membership. However, a few studies presented at the International Congress on Peer Review and Biomedical Publication at Chicago in September 16, 2005 showed that membership is not adversely affected by choosing an open access option. A table of contents comparable to a hard copy of the journal could be e-mailed periodically to all members saving significant resources and costs spent on journal printing and mailing. For those insisting on hard copies of the entire issue, they may be provided with the option with nominal dues. In regard to those countries and communities (without access to computers, internet, and printers), friends and other non-profit organizations can mail the printed PDF files without problems associated with the copyright related restrictions.

There have been some statements regarding exploitation of open access by some industries, especially pharmaceutical companies. Does it matter, who uses it? Many of these commercial entities can see the benefits of open access and may contribute financial resources in the future. The challenge for us is to create different models that explore and tap such resources. When the funding status improves in the near future, as societies actively embrace open access, the editorial and peer-review process which is currently a pro-bono voluntary effort could also be rewarded financially.

Another most important disparity is the present lack of significant direct support from governmental and non-profit funding organizations to the authors publishing in open access. These organizations are spending billions of dollars supporting research, but ultimately allow most of the resulting publications to be lost in traditional non-open access journals. However, there have been some recent efforts to support open access [7-10]. The argument that much research and it's subsequent publications are not generated through grants is a simplification. Almost all research is conducted with public resources and is the result of hard work by academia. It would be unusual to find research generated by publisher's funds for publishing in their journals after paying due honorarium to the researcher. *Thus vast funding is spent at present on the research, but most of such funded research is lost ultimately to non-open access model of publication. If only a tiny fraction of this enormous fund is invested with sincere commitment, most of such research could be rightfully salvaged to be channeled to an open access mode for the general public good in future*.

If funding organizations, whether private, public, non-profit, or governmental, all spend a very small fraction supporting open access publication, the general public would get far greater benefits. If we look at the present scenario, there is not a single funding entity to support general authors publishing their research in open access journals. At the same time universities and other institutions are active in supporting open access on their own. Funding organizations should be encouraged to make grants on an annual basis to support publication by university faculty and society members in open access.

## Peer-reviewer's instructional guide

The retreat also discussed resources for peer-reviewers [11]. Traditionally, the peer-review process has been an amateur voluntary process with only a few journals taking leadership role in extending peer-review guidance material and training opportunities [12]. However, the results of such initiatives have not been evaluated objectively and their effectiveness is not proven. The material available for such instruction is limited and is not specialty or subspecialty specific. An instructional resource for the peer-review process in cytopathology is thus needed.

At this CytoJournal retreat, a task force was coordinated to prepare such a guide specifically for reviewing cytopathology manuscripts. An instructional guide by Callaham et al [13] was identified as a basic model, and we thank Dr. Callaham and the American College of Emergency Physicians for generously providing free CDs of this instructional guide [13] for retreat participants.

The resultant peer reviewer instructional guide for cytopathology manuscripts will be published in an open access format in CytoJournal with an editorial. As a benefit of on-line publication, a multimedia presentation could be included as an 'additional file' for the reader similar to the previous CytoJournal publications [14] and this editorial (Additional files 1 and 2).

## Impact factor

There was considerable interest and concern about the Impact Factor of CytoJournal. Impact Factors are the measure of the importance of scholarly journals. Retreat participants suggested that CytoJournal should give feedback about this to our readers. There is significant information about impact factor on the web [16]. For 2004, the impact factors of traditional existing non-open access cytopathology journals ranged from 0.831 to 1.654 [15]. The impact factor is calculated each year by a commercial company- Thomson Scientific, also known as the Institute for Scientific Information (ISI) [17]. It tracks the citations in selected journals and calculates the impact factor, which are published in the Journal Citation Report [18]. This factor introduced initially by Dr. Garfield [19] has a huge, but controversial, influence on the way researchers perceive and evaluate published scholarly articles [20]. Although this factor was initrially intended to be an objective measure of journal reputability [15], it has been used to measure academic productivity.

The impact factor is calculated based on a three-year period by the following formula:

A = Total number of times the articles published in a two year period (e.g. 2004–05) are cited in ISI tracked journals during the third year (2006)

B = Number of articles published in these two calendar years (e.g. 2004–05)*

The impact factor for the year 2006 equals A divided by B.

* [ISI excludes certain article types (such as news items, correspondence, and errata) from the denominator].

The most commonly cited drawbacks of the impact factor include:

• It does not measure the quality of publication but mostly the quantity.

• Turnover affects the factor. e.g. medical journals generally have higher impact factors than mathematics journals.

• It is more of a popularity gauge than quality.

• The duration of citation is relatively short. Many sentinel articles are cited with significant frequency for longer duration.

• And so on-------[19,21]

Because of this, other alternative ways such as 'h' factor and Y-factor of quantifying and judging publication quality have been suggested [22-24].

As CytoJournal is new, we will have to wait for at least three years from its launch before its Impact Factor can be calculated. BioMed central will activate the mechanism to get the impact factor. Based on preliminary estimation we should have a decent impact factor comparable to other cytopathology journals or even better. Although 'open access' is new, many open access journals already have impact factors, with their pattern of scores similar to that for traditional non-open access journals in respective areas [21,25-27]. CytoJournal was launched in the latter half of 2004 and aims to achieve an impact factor in the near future.

To calculate even an unofficial impact factor, the journal needs to have been published for at least two calendar years (such unofficial factor calculation requires us to know the total number of publications in one year, and the number of times these articles were published in the following year). For example, to calculate the unofficial impact factor for CytoJournal, we would need to count the total number of articles published in 2005 (the first full calendar year of publications by the journal), and the total number of times these articles were cited in the 2006 calendar year. So it will not be possible to calculate even a meaningful unofficial impact factor for CytoJournal before early 2007.

So, until then, please continue to support CytoJournal. We encourage CytoJournal readers to maximize the journal's impact factor by reading and citing it at no cost to you and your colleagues. For those who have already published and cited good high standard articles from CytoJournal, we thank you for your support.

## Readership count of the articles

All journal subscribers do not read all the articles and all the issues. A readership count of articles is in some ways a more objective and real time measure for an individual article, rather than readership numbers of a traditional publication. This monitoring is only possible with digital online events. For CytoJournal, at the time of writing of this editorial, the total hits for the ten most accessed articles was about 28, 000 for its first year in 2005 [28]. All articles are also available via the PubMed Central archive, so the total number of accesses to each article is significantly higher. For a small sub-speciality like cytopathology these are relatively high numbers, and they are rising quickly, as CytoJournal becomes more widely known mainly through word of mouth through readership. *Sign up for the free 'article alert ' on the CytoJournal home page [29] and send the link to all your colleagues to share the benefits with them.*

As a fundamental and important step, please request your respective societies to support open access by organizing a system to allow all members to publish free in CytoJournal as one of the membership benefits of  the society. This will potentially attract more members to the society, and will facilitate open access of cytopathology literature to both contributing authors and readers alike.

In summary, the First CytoJournal Peer-Review Retreat generated excellent discussion and facilitated the development of strategies. *Because of its open access charter, CytoJournal is your scholarly cytopathology journal, peer-reviewed by you, for you. Harvest all its benefits by contributing to it, reading it, and citing it!*

## Supplementary Material

Additional File 1 **Open-Access: A gift to authors and readers- By Lynn Sandweiss**, MPH lsandweiss@mednet.ucla.edu. Chair, *Open-Access *advocacy for CytoJournal, University of California, Los Angeles, USA Click here for file

Additional File 2 **CytoJournal Peer-Review process: goals and resources**- By Vinod B. Shidham, MD, FRCPath, FIAC vshidham@mcw.edu. Executive editor & coeditor-in chief, CytoJournal , Director of Cytopathology Fellowship Training Program & FNAB Service, Director of Fine Needle Aspiration Biopsy Service, Medical College of Wisconsin (MCW), Milwaukee, WI 53226 Click here for file
