# Supplementary material for: First CytoJournal Peer-Reviewer's Retreat in 2006 – Open access, peer-review, and impact factor
Source: Cytojournal. 2006 Mar 27;3:5. doi: 10.1186/1742-6413-3-5 (PMC1421433; doi:10.1186/1742-6413-3-5)

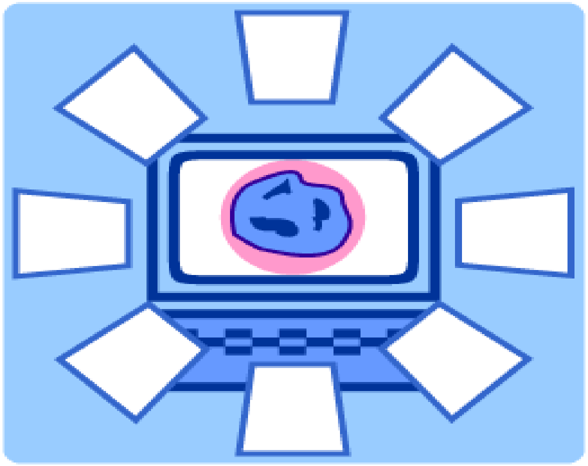

Open-Access: A gift to authors and readers

its strengths and weaknesses

CytoJournal

Advocacy for open access

Open Access

What is Open Access?

- Articles are universally and freely available via the Internet
  - Easily readable format (HTML, PDF)
  - Permanently archived in an internationally recognized open access repository (PubMed Central and others)
- Authors retain Copyright to their article
  - They grant the right to disseminate their article to anyone
  - Integrity of text protected, authorship attributed, correct citation details required

Open Access Calls for Both Free Access and Open Usage

There seems to be a general misunderstanding that the aim of the Open Access movement is *only* to make the scientific research literature free online...

That is the first aim, but it also aims to make it fully usable."

The difference between the two messages is substantial.

Open access Model

- Read it
- Use it
- Cite it

Free the refereed literature now!

American Physical Society

Since 1991

Open Access publishing: the basics

- **Peer Review:** all papers are peer reviewed in the 'traditional' way
- **Secure Archiving:** all articles are permanently archived in PubMed Central
- **Searchable and retrievable:** included in PubMed, Scirus, Google

Peer Review

- Web-based user-friendly system for:
  - Submission
  - Referees to view manuscripts and feed back comments to authors
  - Allows authors to track the progress of their manuscripts
- Rapid process: decision in 7 weeks, on average
- Peer reviewing is done in the traditional sense: 2 peer reviewers plus a statistician if necessary

Open Access publishing: the benefits

- Free Access to anyone with internet access

- Maximum Visibility = Maximum impact!
    - BioMed Central’s papers average >200 downloads per article per month
    - High chance of being cited
    - Speed
    - Online system for submission, peer review and publication
    - Published on the day of acceptance
    - No page constraints
    - Listed without delay in PubMed and deposited in PubMed Central and other secure archives

Open Access publishing: more benefits

- **Authors retain copyright and are free to post papers on the web and distribute it to colleagues**
- **Lower cost to libraries and institutions**
- **Authors can access download statistics which are displayed on the site for the past 30 days and for the most-viewed/accessed articles**

Has anybody read it?

- **Most papers on BioMed Central average >400 hits / month**
- **Not unusual to get 2-4000 hits / month**
- **http://www.biomedcentral.com/my/**

BioMed Central

BioMed Central Open Access Charter

The author(s) or copyright owner(s) irrevocably grant(s) to any third party, in advance and in perpetuity, the right to use, reproduce or disseminate the research article in its entirety or in part,

Provided no substantive errors are introduced in the process,

Proper attribution of authorship & correct citation details are given,

And that the bibliographic details are not changed. If the article is reproduced or disseminated in part, this must be clearly and unequivocally indicated.

BioMed Central, more…

- BioMed Central is committed permanently to maintaining this open access publishing policy, in all eventualities, including any future changes in ownership.
- Every peer-reviewed research article appearing in any journal published by BioMed Central is 'open access', meaning that:
- The article is universally and freely accessible via the Internet
- In an easily readable format and deposited immediately upon publication

BioMed Central's Journals

- In at least one widely and internationally recognized open access repository (such as PubMed Central)
- Over 130 open access journals in total in all areas of biology and medicine
- Access to all peer-reviewed research content is free

How is Open Access achieved at BioMed Central?

- Removing subscription charges and introducing article processing charges, while allowing authors to keep the copyright to their article
- Article processing charge per published article
  - Waivers are available
  - No color charges or other ‘hidden’ charges
- Model further financed by some advertising

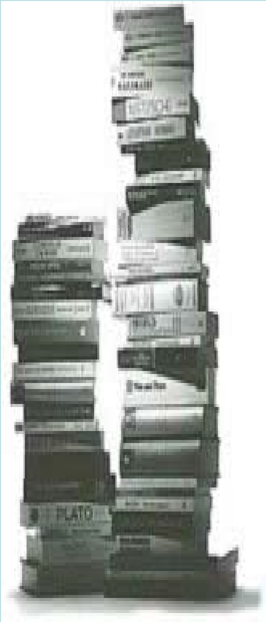

Print Journals

The print journal process

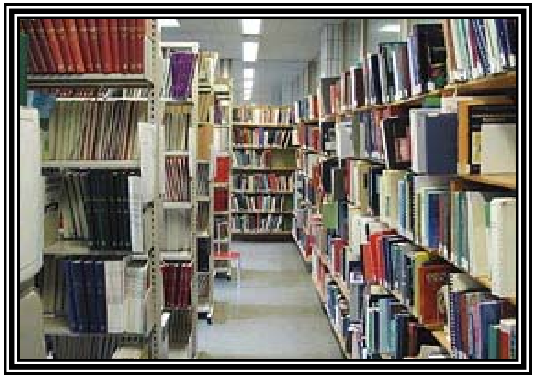

- The <u>scientific community</u>:
  - Conducts, analyzes, writes up and peer reviews research
  - Sometimes also funds research
- <u>Print journals</u>:
  - Sell freely donated information by restricting access to it
  - Editorial policy often influenced by advertisers
- The <u>public</u>:
  - Funds research through tax $$ but has limited access to results (abstracts through PubMed)

Commerce and scientific publishing

- Privatization of the journals for the sake of commercial interests has damaged the philanthropic viewpoints of science for all and everywhere for many decades.

- Commercial interests support the advertising of their products and services in journals. From their perspective, journal articles appear between advertisements to entice the reader to see the ads.

- We read the journals for the articles, not the advertisements, and don't want editorial policy being dictated or influenced in an way by corporate interests.

Comparisons

Advantages of open access

- Free access
- Copyright retained
- Rapid publication (4-10 weeks)
- Trackable readership
- Include data/graphs/tables/photomicrographs/videoclips
- Secure archiving
- Easy online submission
- Efficient online transmission

Disadvantages of open access

- At this point in time, readership likely not as great as that of print journals
  - This point is debatable as there is no way to track readership of print journals
  - Subscribership is traceable
  - Readership of individual articles is not
- Open access literature currently represents only 1% of all published literature

Comparison: free access versus print

- Peer reviewed
- Indexed by Medline
- Easy online submission
- Open access
- Copyright retained
- Rapid publication (4 – 10 weeks)

- Peer reviewed
- Indexed by Medline
- Standard postal submission
- Pay by subscription for full text
- Copyright property of journal
- Non-rapid publication (months to years)

Comparison: free access versus print

- Secure archiving
- Track readership
- Include data/charts/ photomicrographs
- Videoclips accepted

- Journal secures copyright
- Tracking of citations only (through Web of Science)
- Limited space for charts/ graphs/ photomicrographs
- No videoclips

Today's (soon yesterday's) **print-derived model**

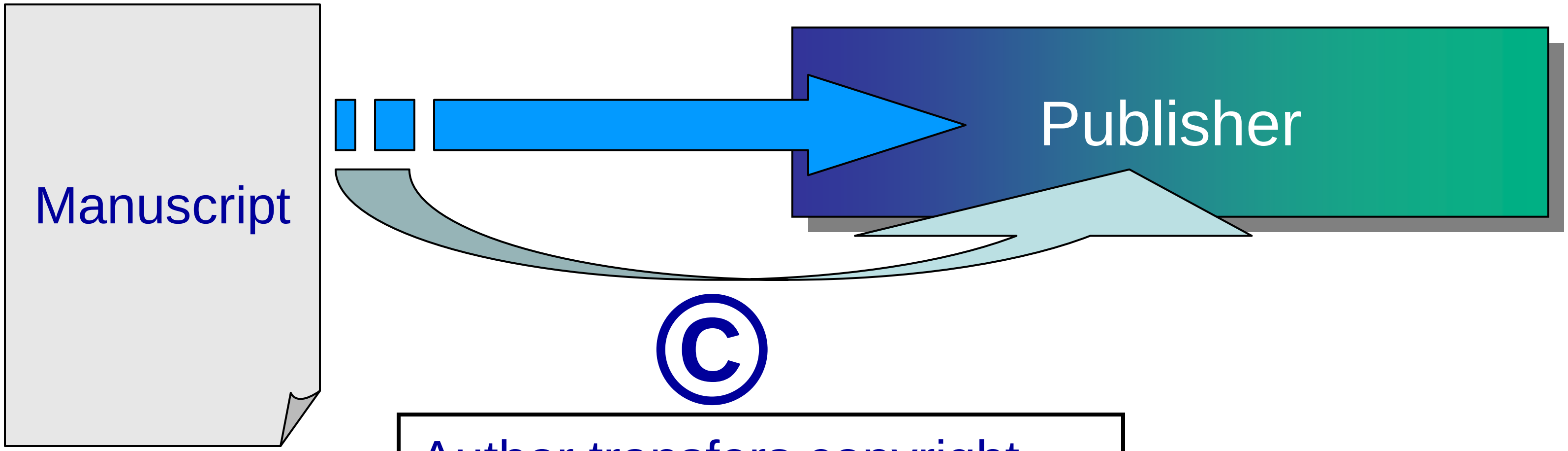

Author transfers copyright
or exclusive publishing rights

Result:

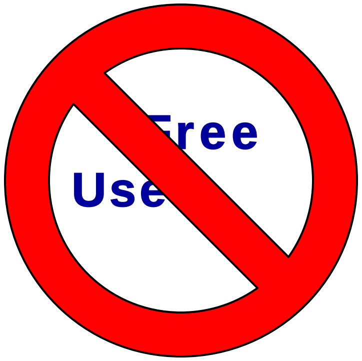

Only those who can afford expensive subscriptions or licences have access

Tomorrow's (today's at BioMed Central) online-based model:

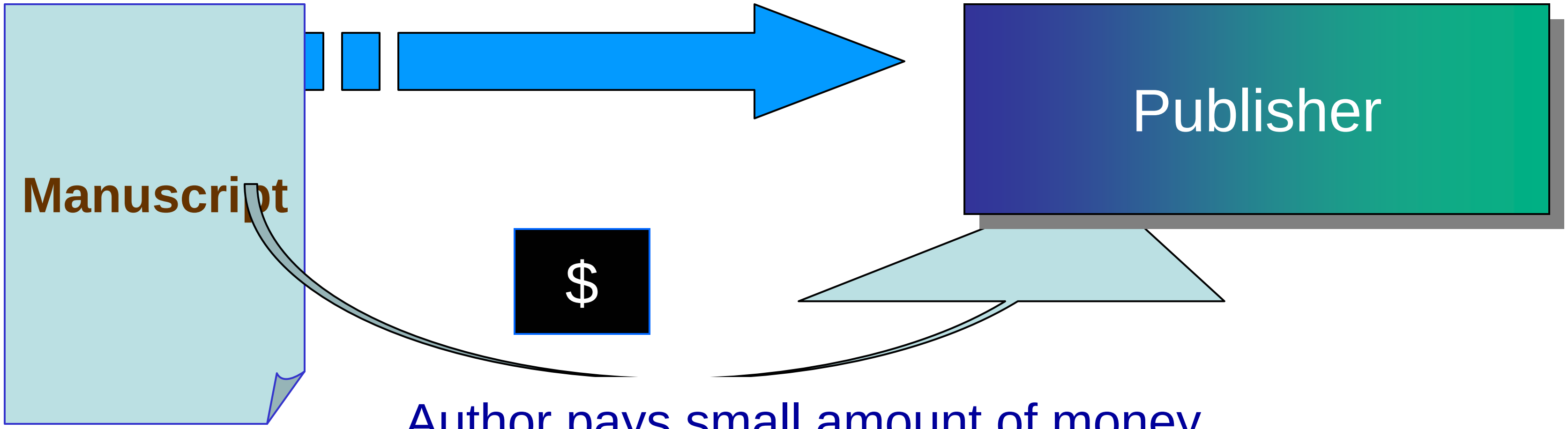

Author pays small amount of money
or rather, institution pays on author's behalf

Result:

OPEN ACCESS

Everyone has access
All use is fair use

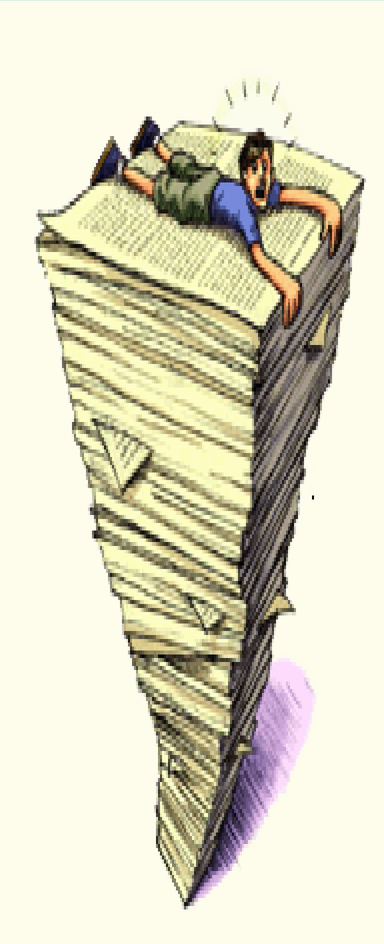

Problems with print journals

System unjust

- The scientific community does the work
- Publishing companies profit
- For example:
  - Why does an author have to buy back the right to distribute his/her own research? (e.g. $600 for 200 reprints)

Restricted access

- **How does the scientific literature help:**
  - **A community pathologist seeking information about newer ancillary technologies**
  - **A pathologist in a developing country (subscriptions typically 25 – 50% of median yearly wage)**?

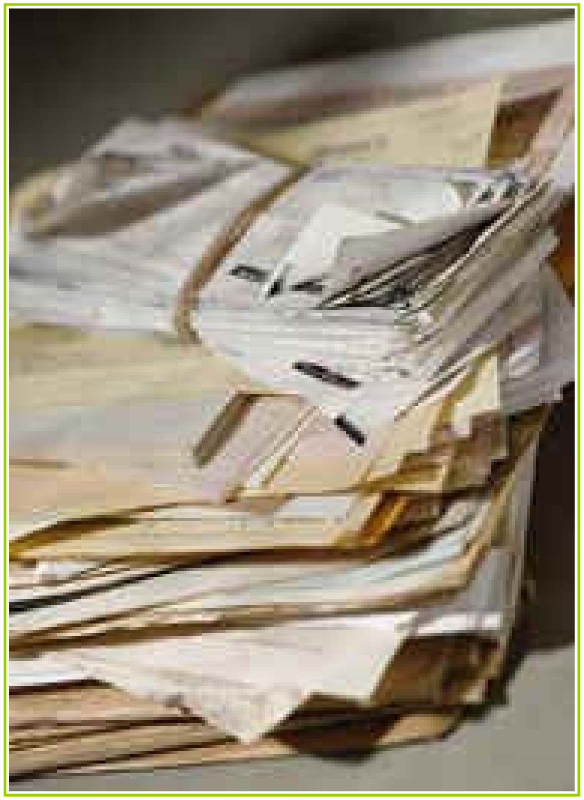

Time to print

- **Journals must wait for an appropriate slot to become available given limited journal space**

- **Typical turnaround between submission and publication 6 – 12 months**

Dissemination inefficient

Photocopying of journals stored in a library (or posting of reprints) is time consuming, expensive and inefficient

Following up citations is time consuming

No connection with subsequent research, comments, errors etc.

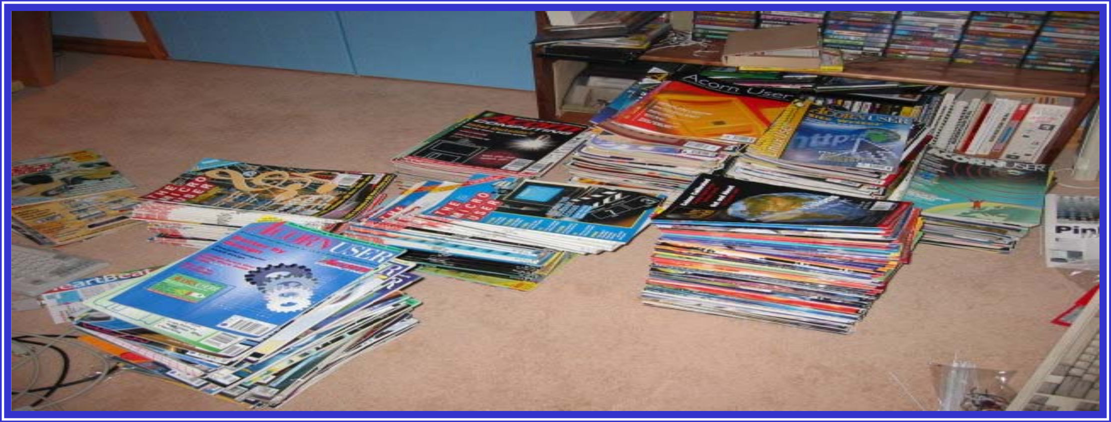

Paper publishing extremely expensive (esp. for forests)

- The scientific community pays for each article published by buying subscriptions
- Cost per article increased 2.6 fold from 1975 – 1995 adjusted for inflation
- Even institutions find it increasingly difficult to provide access to all the journals that their communities need

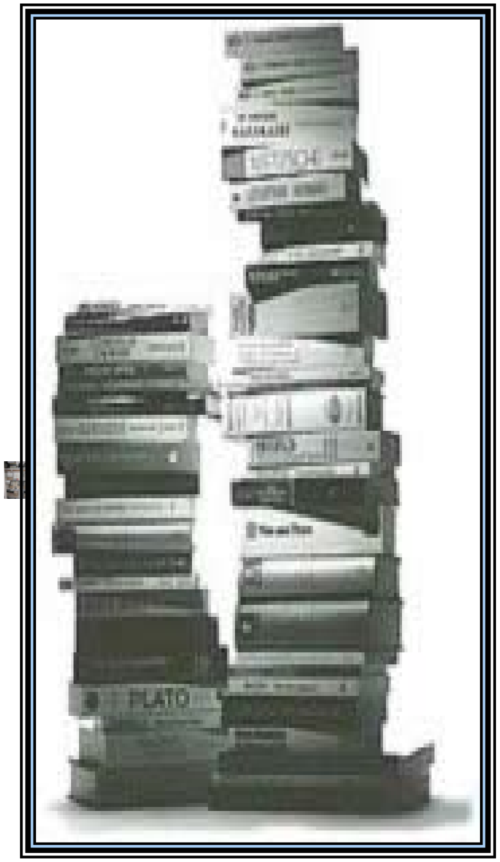

Environmentally unsustainable

- Journal of Clinical Oncology has 26,000 subscribers, 24 times / year
- 16 stacks of journals each as high as the Empire State Building
- Must be transported and filed

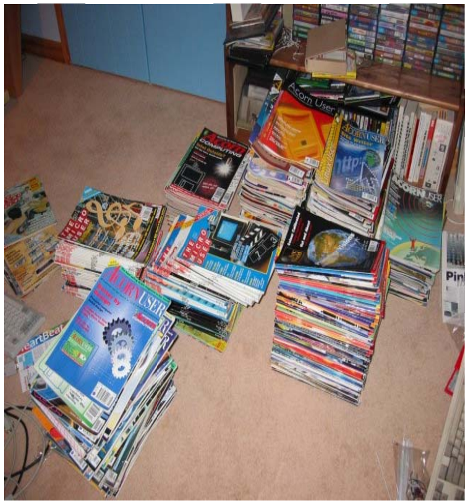

In summary:

- Paper journals are:
  - Inequitable (positive bias?)
  - Inaccessible
  - Inefficient
  - Extortionate
  - Inexpedient
  - Unenvironmental
  - Dispensable?

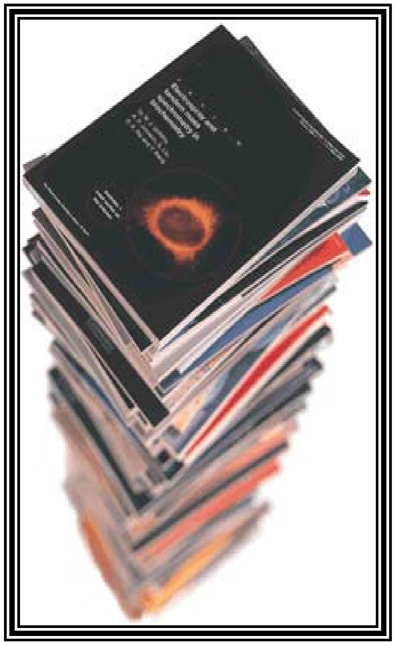

Do we need paper journals?

- 20 years ago, only a journal could disseminate information.

- Journals provided a necessary service

- Do we need paper journals in the Internet age?

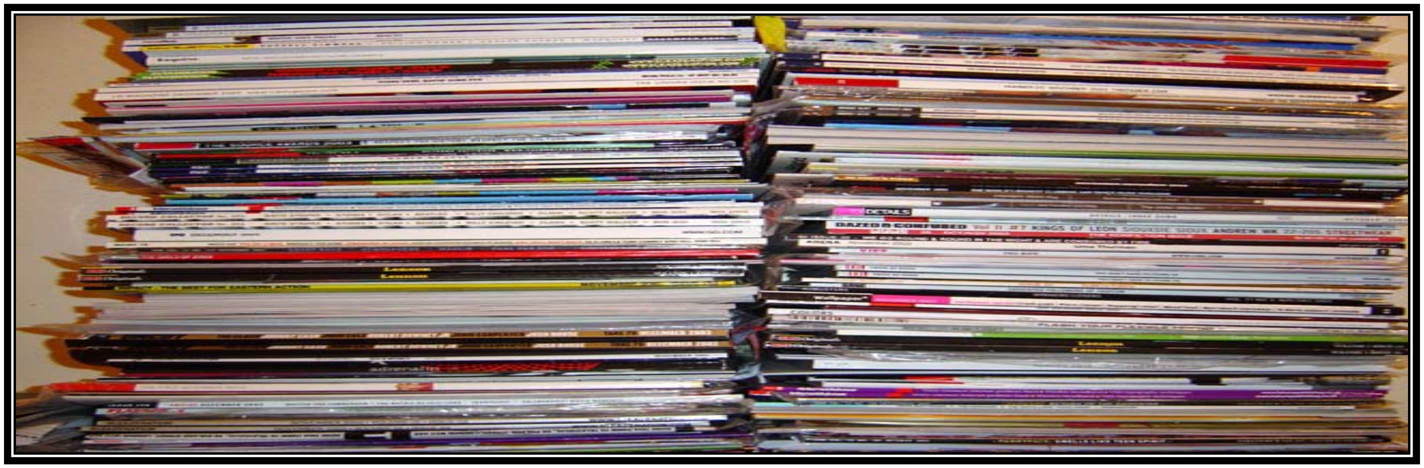

Supplement: Additional File 1 — Open-Access: A gift to authors and readers- By Lynn Sandweiss, MPH lsandweiss@mednet.ucla.edu. Chair, Open-Access advocacy for CytoJournal, University of California, Los Angeles, USA [file 1742-6413-3-5-S1.pdf]
